# Potential of Superhydrophobic Surface for Blood-Contacting Medical Devices

**DOI:** 10.3390/ijms22073341

**Published:** 2021-03-24

**Authors:** Xun Hui Wu, Yun Khoon Liew, Chun-Wai Mai, Yoon Yee Then

**Affiliations:** 1 School of Postgraduate Studies, International Medical University, Kuala Lumpur 57000, Malaysia; XunHui.Wu@outlook.com; 2 Department of Life Sciences, School of Pharmacy, International Medical University, Kuala Lumpur 57000, Malaysia; LiewYunKhoon@imu.edu.my; 3 Department of Pharmaceutical Chemistry, School of Pharmacy, International Medical University, Kuala Lumpur 57000, Malaysia; chunwai_mai@imu.edu.my

**Keywords:** superhydrophobic, medical device, antihemolytic, antithrombotic, antibacterial, anti-biofouling, blood compatible

## Abstract

Medical devices are indispensable in the healthcare setting, ranging from diagnostic tools to therapeutic instruments, and even supporting equipment. However, these medical devices may be associated with life-threatening complications when exposed to blood. To date, medical device-related infections have been a major drawback causing high mortality. Device-induced hemolysis, albeit often neglected, results in negative impacts, including thrombotic events. Various strategies have been approached to overcome these issues, but the outcomes are yet to be considered as successful. Recently, superhydrophobic materials or coatings have been brought to attention in various fields. Superhydrophobic surfaces are proposed to be ideal blood-compatible biomaterials attributed to their beneficial characteristics. Reports have substantiated the blood repellence of a superhydrophobic surface, which helps to prevent damage on blood cells upon cell–surface interaction, thereby alleviating subsequent complications. The anti-biofouling effect of superhydrophobic surfaces is also desired in medical devices as it resists the adhesion of organic substances, such as blood cells and microorganisms. In this review, we will focus on the discussion about the potential contribution of superhydrophobic surfaces on enhancing the hemocompatibility of blood-contacting medical devices.

## 1. Introduction

Materials with superhydrophobic properties have been receiving hefty attention since their discovery. Superhydrophobic surfaces have been vastly studied and incorporated into applications across various fields. In light of their non-wetting behavior, superhydrophobic properties have been highlighted in the development of biomaterial for medical devices. Blood-contacting medical devices are commonly seen in medical and healthcare settings, either for their diagnostic purpose or treatment purpose. Medical implants and external medical devices, including stents, vascular graft, heart valve, artificial kidney, pacemaker, guidewires, extracorporeal circulation, tubing, and catheters are examples of medical devices that are particularly close in contact with blood during their applications. Despite their inevitable role in clinical practice, blood-contacting medical devices are associated with thrombotic complications [1,2]. Furthermore, hemolysis and device-related infection are also often the major drawback of blood-contacting medical devices [3,4,5].

Many approaches have been taken in order to circumvent these problems. Nonetheless, blood compatibility remains a long-existing challenge in developing biomaterials for blood-contacting medical devices. Significant blood-compatible biomaterials suitable for medical devices are difficult to be forged as the mechanism of adhesion of blood cells and microorganisms on the surface are complex and multifactorial [6]. Boundary condition also takes part in affecting the interaction between molecules and surface. Superhydrophobic surface depicts a promising result in enhancing the blood compatibility of medical devices. The hierarchical structures on superhydrophobic surfaces have substantiated to offer good hemocompatibility by diminishing adhesion force. Blood travels across superhydrophobic surfaces with a greater velocity on the boundary layer, thus reducing the collision frequency of blood cells with the surface [7]. Consequently, this results in lesser adhesion and deformation of blood cells. In this review, we will discuss the role of superhydrophobic surfaces in mitigating the existing issues of blood-contacting medical devices.

## 2. Characteristic of Superhydrophobic Surface

The superhydrophobic surface is defined by a surface exhibiting an apparent contact angle greater than 150°, contact angle hysteresis lesser than 10°, and sliding angle lesser than 10° [6,8]. Contact angle is the angle depicted by water droplet at the contact line when it comes in contact with the solid surface. It is commonly used as a measure of wettability. Contact angle hysteresis is the difference between the advancing contact angle and receding contact angle. It is used to evaluate the repellency of the solid surface towards the water droplet. The advancing contact angle is usually greater than the receding contact angle. The lesser the difference between these angles, the greater the non-stickiness of a droplet on the surface. In other words, superhydrophobic surface with a high apparent contact angle and low contact angle hysteresis allows the water droplet to roll off easily [9]. On the other hand, if the high apparent contact angle is accompanied by high contact angle hysteresis, this situation is known as the rose petal effect, whereby the droplet is pinned on the surface [10,11,12,13]. The differences between these two superhydrophobic wetting conditions will be further discussed in this section.

**Figure 1:**
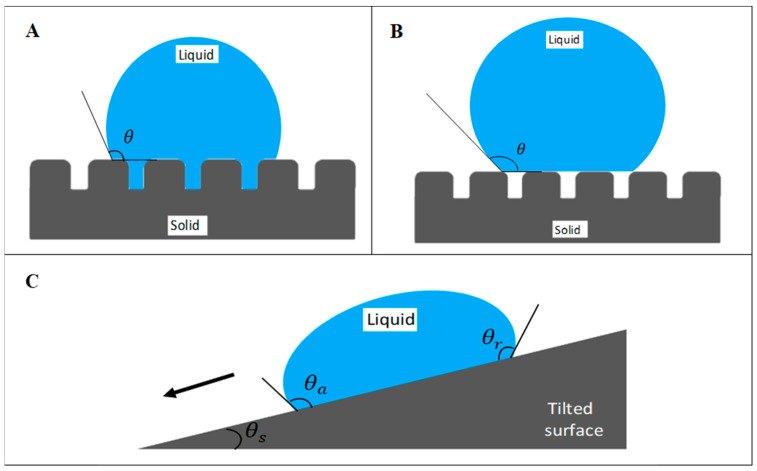
Wetting states of surface based on (**A**) Wenzel’s model and (**B**) Cassie–Baxter’s model. (**C**) Schematic of a water droplet slides off a tilted surface. *θ* represents the apparent contact angle, which measures the wettability of the surface by a liquid droplet. *θa*, *θr*, and *θs* represent advancing contact angle, receding contact angle, and sliding angle, respectively.

Superhydrophobicity is elucidated by two physical principles: low surface energy and high surface roughness. Both surface chemical composition and surface morphology are the major factors in interfering with the interaction between liquid and solid interface. Surface energy influences the adhesion of substances, including fluid and microorganisms, on the surface. Low surface energy reduces the work of adhesion and therefore increases the water repellency. According to Wendel’s model and Cassie–Baxter’s model, surface roughness plays a critical role in wettability (Figure 1). The micro/nanostructure of the surface allows entrapment of air layer beneath the contacting liquid, therefore reducing the contact area between the liquid and solid surface [9,13,14]. Besides, the presence of air pockets on the surface endows lower frictional drag, which allows effective fluid flow [15]. Hence, the collaboration of surface roughness and low surface energy of the fluorinated polymers are to be highlighted in superhydrophobicity.

As the topography of the nano-/micro-scale roughened surface and/or chemically heterogeneous surface are not able to be viewed under regular optical means, hence the wetting of these surfaces is characterized by apparent contact angle [16,17]. However, pure Wenzel and Cassie–Baxter wetting states are rare to be observed in nature. Instead, a condition known as the mixed-wetting state is more common to be seen, whereby the droplet is partially supported by the air as well as the rough chemically homogenous surface [18]. It is noteworthy that the Cassie state is metastable, even on a rough surface. The entrapped air may escape and transition into the Wenzel state.

In order to maintain the thermodynamic stability of the Cassie state, the critical angle of the air entrapped below the water droplet must be small [19]. A thermodynamic equilibrium of the liquid/solid/vapor system must be attained to generate an ideal wetting surface. Air-trapping which occurs in the Cassie–Baxter state, as aforementioned, is found to be metastable as it gradually shifts to the Wenzel state [16,20,21]. Therefore, maintaining the entrapment of air pockets is important for the Cassie regime as the irreversible transition can be due to the invasion, such as condensation or evaporation of water droplets, or through external pressure [21]. The superhydrophobic surface loses its water-repellent properties when the air gap between the structured surface is filled with water. The Cassie–Baxter state demonstrated weak drop adhesion and reduced friction properties, therefore providing the “Lotus effect” or self-cleaning properties. Remarkably, shifting from the Cassie–Baxter state to the Wenzel state affects the drag reduction by altering the flow rate [15]. Besides, the surface loses its self-cleaning effect and promotes adhesion of the water droplets instead when the wetting state shifts from the Cassie–Baxter to the Wenzel regime [21,22]. The Wenzel state allows the water droplet to pin on the surface (known as the rose petal effect) while exhibiting higher contact angle hysteresis, as compared to the Cassie state, which exhibits low contact angle hysteresis [20]. The Wenzel state promotes the pinning of the droplet on the surface due to the complete wetting on the ground level of texture [21,22]. Based on the distinction between both wetting states, the Cassie–Baxter regime is hence preferable and should be taken into consideration during the development and fabrication of superhydrophobic surfaces in medical devices.

## 3. Development of Superhydrophobic Surface

The principles of superhydrophobic surface are inspired by nature (Table 1). The most classic example would be the lotus leaf. Besides having a high contact angle, the hierarchical structure on the lotus leaf endows its superhydrophobicity. The term “Lotus effect” is derived from its self-cleaning behavior. Non-adhesive lotus leaf surface is able to repel water droplets and allows them to easily slide off. Further examples of superhydrophobic surfaces that can be found in nature are available in other studies [23,24]. While producing superhydrophobic surfaces by mimicking lotus leaf is the most common approach among previous studies, endothelial cells lining of a human blood vessel is also another biomimetic structural superhydrophobic surface [25].

Artificial superhydrophobic surfaces can be produced via different routes and techniques, including surface treatment, changing their surface composition, or altering their surface texture (Table 2). Some materials hitherto less hydrophobic can be transformed into superhydrophobic via modifications. For example, hydrophilic polyvinyl alcohol (PVA) film (contact angle 72.1°) was transformed into a superhydrophobic film (contact angle 171.2°) by adding PVA nanofibers [30]. The addition of silicon nanofibers increases the contact angle of a hydrogen-terminated silicon surface from 74° to 160° [31]. Introduction of nanostructures and fluorinated alkyl side chains on smooth poly(carbonate urethane) film increases its contact angle from 109.1° to 163.6° [32]. The progress of developing an efficient superhydrophobic surface during the past few years has been reviewed. Different types of approaches to fabricate superhydrophobic surfaces have been analyzed [9,13,33,34].

As aforementioned, surface morphology can act as a significant factor in influencing superhydrophobicity. Surface roughness endows a higher apparent contact angle, therefore enhancing the wettability of the surface. As described by Cassie and Baxter, the surface texture creates air pockets between the protrusions. These air pockets limit the contact point between the liquid and solid surface. Modifying surface texture is a simple and economical way to enhance superhydrophobic properties. With the help of modern technology, surface roughness can be increased, for instance, through oxygen plasma treatment or via a simple lithography technique known as the nanoimprint process to create nanostructures on the surfaces [43]. On the other hand, modification of the surface chemical composition is another alternative to prepare a superhydrophobic surface [44].

The height of protrusion that made up the hierarchical patterned surface acts as a factor in swaying the self-cleaning ability of the surface. As the protrusion is sufficiently high to allow the contaminant partially to fill in between them, or the contaminant is small enough to penetrate the coating, it will produce a surface with increased roll-off angle, whereby the self-cleaning effect is futile. Micro/nanopatterned surfaces with pore sizes below 500 nm withstand most kinds of particulate contamination [45]. On the other hand, another study indicated that microstructured surfaces with protrusions size range below 5 µm can be cleaned easily by fog, as the size of water droplets within the fog appears to be higher than that [46].

Self-cleaning can be enhanced by introducing structures with appropriate height and width on the surface. Low protrusion height and wide protrusion size endow a larger contact area for the contaminant to attach, which causes higher difficulty in removal. As the distance between the protrusion is sufficient to disable the filling of the contaminant in between, and at the same time, the water droplets elevated by the protrusions confer a low roll-off angle, the self-cleaning effect is said to be plausible [46].

Superhydrophobic properties can be enhanced by altering the surface structure while maintaining the chemical composition of the target surface, as reported by Mao et al. and Ryu et al. [38,40]. Both studies transformed smooth polymer surfaces into an ideal blood-contacting superhydrophobic biomaterial. Mao et al. fabricated polystyrene nanotube films by mimicking the structure of lotus leaf, whereas Ryu et al. performed plasma-etching on polytetrafluoroethylene. The superhydrophobic polymer surfaces exhibit high contact angles, 151° and 171.4°, respectively. In addition to their self-cleaning effect, the surface also demonstrated high durability, although negligible deformation on the nanoscale structure surface was observed under SEM after prolonged usage and exposure to air. Importantly, the nano/microstructures of superhydrophobic films contribute to the low adhesion of blood cells and platelets. On the other hand, Helmer et al. fabricated a transparent fluorinated polymer foam via a simple one-step photoinitiated radical polymerization [36]. The nano/microstructure of the foam-like polymer provides its superhydrophobic characteristic with a contact angle of 163.7° and a contact angle hysteresis of 6.1°. This easy-to-fabricate material is resistant to abrasion as well. The hierarchical surface roughness is substantiated to increase the superhydrophobic effect, thus improving the anticoagulation and blood-repellent effects.

## 4. Promising Effect of Superhydrophobic Surface on Medical Devices

### 4.1. Antihemolytic Effect of Superhydrophobic Surface

Different from water, a Newtonian fluid, blood is a shear-thinning fluid. Shear stress causes red blood cells to rupture or endure shape alteration. The viscosity of blood is consequently reduced due to the reduction in red blood cell concentration. This phenomenon bestows its non-Newtonian behavior. Hemolysis is remarkably a serious complication encountered by blood-contacting medical devices. Hemolysis causes the release of hemoglobin (Hb) and adenosine diphosphate (ADP), which then stimulates the activation of platelets. The released Hb inhibits nitric oxide to suppress platelet activation [47]. The sequences of action may lead to a life-threatening thrombotic event. Poor selectivity in lab-on-a-chip applications might also be a result of device-related hemolysis.

The mechanism of device-related hemolysis alluded are damages resulting from either the shear forces in the blood flow or the physical contact between the blood cells and surface. The intensity of shear stress acting on the blood cells depends on the position and orientation of the cells in the shear field, as well as the proximity of other cells and components within the blood. If substantial shearing force is applied, it may induce an irreversible deformation in the membrane structure of red blood cells. Blood–surface interaction should also be taken into consideration as the factor of hemolysis [48].

In contrast to the no-slip boundary, where blood travels at zero velocity, superhydrophobic surface lubricates the fluid flow by introducing an effective slip boundary by reducing the drag force. The drag reduction attributes to the stable pockets of air between the solid–liquid boundary reduces. As the skin friction is reduced, blood flow will be enhanced by accelerating slip velocity with minimum shear stress [49]. This postulation concurred with the study by Hoshian et al. [6]. Shear force and viscous drag of blood flow across the superhydrophobic surface are greatly reduced due to the reduction in hydraulic resistance. The superhydrophobic surface exhibits excellent blood repellence and enables blood transportation without macroscopic losses. Blood droplet slides off the superhydrophobic surface and allows easy removal by gentle washing without any residues observed.

On the other hand, Ou et al. suggested that the air layers trapped by the hydrophobic surface reduce the contact area between liquid and solid surface. Therefore, this results in diminished shear stress experienced by fluids flowing past near-superhydrophobic/superhydrophobic surfaces [50]. Reduction in hemolysis rate is observed when blood flow is through a poly(vinyl chloride) pipe coated with a near-superhydrophobic surface. This near-superhydrophobic surface allowed the red blood cells to flow past the fluid–solid interfaces with diminished shear stress despite the presence of a no-slip boundary condition [51].

**Figure 2:**
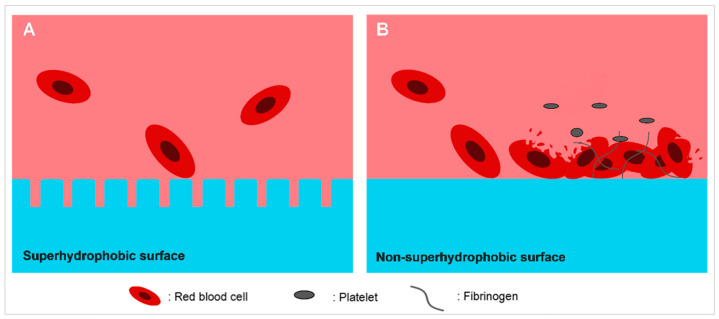
Comparison between the hemolytic effect of (**A**) superhydrophobic surface and (**B**) non-superhydrophobic surface. Superhydrophobic surface repels the red blood cells, leaving the blood cells unharmed due to the structured surface and low surface energy. On the other hand, red blood cells tend to adhere to the non-superhydrophobic surface, denature and promote the adhesion of clotting agents, consequently leading to thrombosis and entrapment of microbial cells.

Red blood cells adsorb on the surface of medical devices upon contacting, and this further leads to crowding of the blood cells. Consequently, the red blood cells are vulnerable to lysis even under osmotic condition (Figure 2). Reports implied that low surface-free energy reduces the collision between blood cells and the surface, whereas multiscale structured surface reduces the contact area between blood cells and surface [7,52,53]. Consequently, the probability of red blood cells denaturation upon cell–surface interaction will be reduced.

### 4.2. Antithrombotic Effect of Superhydrophobic Surface

Medical device-induced thrombosis is a major concern that must be tackled in any event. The adsorption of plasma proteins on the surface occurs as soon as the blood physically contacts with the medical device. Subsequently, a series of mechanisms will occur and consequently leads to blood coagulation [33,47]. These platelets promote the activation of nearby platelets and further lead to the adhesion and aggregation of platelets. Consequently, these aggregates are then stabilized by fibrin to form thrombi. Blood coagulation cascade can be triggered via the intrinsic pathway as well as the extrinsic pathway. Both pathways result in thrombus formation. These thrombotic complications are often found on extracorporeal circuits, vascular graft, central venous catheter, mechanical heart valve, dwelling devices, and implants as the mechanism of clotting in medical devices is mediated via the intrinsic pathway [1].

To date, various approaches have been studied to mitigate the issue of clotting due to the adhesion of fibrin and platelets in the blood flow against the surface of medical devices. Systemic anticoagulant and/or antiplatelet agents have been co-administered to reduce clots formation in patients who are wielding the medical devices [54,55]. Unfortunately, the administration of anticoagulants requires proper monitoring as a preventive measure for the bleeding complication to ensure patient safety. Besides, a previous study implied that not every anticoagulant is suitable to be used in patients with an indwelling device as they do not provide beneficial results [56]. On the other hand, immobilization of heparin on blood-contacting surfaces has been noted to reduce thrombosis as well as lower anticoagulant administration [3,57,58]. In addition to the antithrombotic effect, heparin-conjugated surface possesses enhanced hydrophobicity, as compared to the untreated surface, by exhibiting lotus-like microstructure [59]. However, heparin-coated materials may cause leaching issues, which reduces the anticoagulation effect over time [3,57]. As aforementioned, these strategies have their own limitations and drawbacks. Hence, superhydrophobic biomaterials are proposed to be a promising approach in reducing thrombosis of medical devices.

Fibrinogen is the first blood plasma protein to adsorb on the contacting surface [1]. Therefore, limiting the attachment of fibrinogen may mitigate the consequent complication of thrombus formation. Modification of titanium surface to form a nanotextured and low surface energy surface demonstrated reduced adsorption of fibrinogen from platelet-rich plasma [60]. The presence of air pockets allows minimal contact between the blood and the nanotextured superhydrophobic surface. Besides, the superhydrophobic surface endows a slip boundary which facilitates the blood flow without promoting cell–surface interaction [49]. The superhydrophobic surface tends to repel the blood cells as they directly contact with each other, therefore the physical damage due to cell–surface interaction can be reduced. This mechanism limits the cell molecules and proteins from adhering to the surface, hence reducing the likelihood of thrombotic events.

Anti-adhesion features of the superhydrophobic surface have received vast attention from researchers. Superhydrophobic surfaces have been developed, for instance, by mimicking multiscale micro/nanostructures of the endothelial surface of natural blood vessels. The anti-adhesion features are attributed to the hierarchical nanostructure of the superhydrophobic surface, which offers a smaller contact point for the platelet to anchor on [61]. Previous study by Koc et al. reported the resistance of nanostructured fluorinated surface toward protein adhesion due to its increased hydrophobicity and reduced interfacial surface area available for protein adsorption. Instead of complete inhibition of cell adhesion, the superhydrophobic surface promotes desorption and detachment [62]. In concurrence with the study by Lim et al., reduction in the effective contact area between the blood and the surface tends to suppress blood clotting [33].

**Figure 3:**
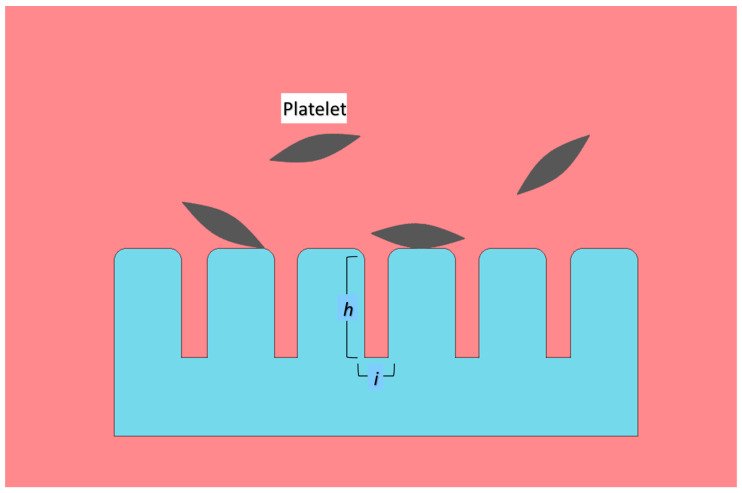
Structured surface embedded with protrusions. The protrusions increase surface roughness, thus providing resistance to cell adhesion. *h* and *i* represent height and interspacing area, respectively. The roughness of the surface depends on the size of protrusions. The anti-adhesion abilities are to be adjusted by altering the height of protrusions and/or interspacing between them.

Structured surfaces are normally roughened by protrusions and the height of protrusions is correlated with shear stress. Under the blood flow condition, the shear stress on top of the protrusion is higher than that on the ground area. The shear stress difference suppresses platelet adhesion by reducing the transfer of platelets from the upper region to the lower region. Therefore, there is lesser platelet adhesion on the ground area, while blood flow-induced desorption of platelets on the tip of protrusions. Besides the height of protrusion, the interspacing area between the protrusion was also reported to be a crucial factor in platelet adhesion (Figure 3). The shear stress difference is lower as the interspacing between the protrusion is reduced [63]. Extreme roughness of the surface (<50 nm), on the contrary, has little influence on platelet adhesion. As the surface features are smaller than the dimension of pseudopods of platelet cells, the surface is considered smooth enough for the impact of roughness to be negligible [64].

In short, the superhydrophobic surface is substantiated to be less thrombogenic than the untreated smooth surface. Besides, the superhydrophobic surface appears to be an encouraging method to reduce thrombosis, as compared to surface modification with immobilization of heparin or other anticoagulants. The superhydrophobic surface reduces the fusion of platelets and blood cells, thus obstructing the thrombi formation. The hemophobic properties of superhydrophobic surfaces drastically reduce the tendency of blood clotting. In light of these characteristics, superhydrophobic surfaces offer a potential solution to the long-standing problem of thrombogenicity in blood-related bio-medical devices and blood vessel implants.

### 4.3. Antimicrobial and Antifouling Properties of Superhydrophobic Surface

Device-related infection, for instance, catheter-related bloodstream infection, has been a major health risk among hospitalized patients, especially those who received indwelling medical devices. When the medical devices are exposed to blood, microorganisms in the blood flow are given the opportunity to adhere to the surface. The bacteria initially attached to the surface begin to form microcolonies. The microcolonies then differentiate into thick structured biofilms which are highly resistant to antimicrobial agents. Biofilms are primarily composed of proteins and polysaccharides, acting as a protective barrier for the bacteria cells community from extreme environments and various antibiotics [65,66,67,68].

Many approaches are being studied to reduce the mortality caused by this complication, including biocide coatings, antibiotic release from surfaces, and materials that promote non-pathogenic bacterial adhesion. However, these approaches have yet to be proven to successfully tackle the issues [42,69]. Antimicrobial material, for instance, which releases biocide to kill the microorganism present on the medical devices, has been a fruitful approach. However, the bactericidal activity will taper off once the anti-infective compounds subside and hit below lethal concentrations, which may pose a threat instead. Administration of antibiotics at sublethal doses has been shown to accelerate resistance pathways and biofilm formation [58]. Besides, the incorporation of biocides agents in medical devices may result in bacterial resistance and induce toxicity [69]. On the other hand, superhydrophobic surfaces or materials have been recently reported to act as a potential strategy. Superhydrophobic surfaces exhibit the “Lotus Effect”, which can be elucidated by their anti-biofouling properties, reducing protein adsorption [8,70]. Due to the low adhesion force, fluid travels through superhydrophobic surfaces without leaving any tracks, and at the same time, tends to pick up substances along the way.

The rough topography provides a limited contact area between microorganisms and the superhydrophobic surface, reducing the chances for biofouling. Surfaces having protrusions with proper height at proper intervals can trap an adequate amount of air in the surface structure. These entrapped airs are responsible for the low sliding angle, and hence self-cleaning. A higher trapped-air ratio in between solid and liquid interface significantly allows greater antifouling properties [18]. The presence of local curvature by the protrusions reduces the anchoring points for bacteria cells. On the other hand, the spacing between the structured surface smaller than the size of bacteria cells has a strong likelihood to prevent anchoring of the bacteria [71].

In addition, the correlation between surface energy and bacteria adhesion is reported. Surface-free energy around 25 mN/m potentially mitigates the viability of bacteria [72]. Fluorinated coatings have been reported to prevent fouling effectively due to the modification of surface-free energy to around 20–30 mN/m [73]. Another study on fluorinated porous surface reported lesser bacterial adhesion, as compared to the random distribution of bacteria adhesion on the smooth surface. Fluorination alters the work of adhesion by lowering the surface energy, and thus facilitates the self-cleaning effect. Bacterial adhesion is proposed to be relatable with the spatial distribution of roughening structures and macro/microscopic patterns on surfaces [39].

Jenkins et al. recently reported the bactericidal effect of nanotextured materials. The nanostructured surfaces exhibit antibacterial activity via mechanical rupture of bacteria cell. The study denoted that surface modification enhanced the photoactive antimicrobial activity of the titanium dioxide surface. However, further enhanced via nanopillar structures on titanium surface induce envelope deformation and penetration of bacteria. The nanopillar surface structures induce oxidative stress response within the bacteria via increased reactive oxygen species, and therefore leads to deformation of bacterial envelope morphology [74]. Another corresponding study by Francolini et al. endorses the antibacterial activity of titanium dioxide via oxidative stress induced by photogenerated reactive oxygen species [75].

Bartlet et al. fabricated a superhydrophobic titania nanotube array via adonization and chemical vapor deposition of (heptadecafluoro-1,1,2,2-tetrahydrodecyl)trichlorosilane [42]. The titania surface exhibits superhydrophobic characteristics including contact angle, contact angle hysteresis, and roll-off angles of 164°, 3°, and 3°, respectively. When observed under fluorescence microscopy, a lower amount of Gram-positive and Gram-negative bacteria are attached to the titania surface without biofilm formation. However, it should be noted that the superhydrophobicity of the surface does not repel bacteria completely. Instead, the bacteria adhered within the grooves and/or spaces between the nanotube arrays.

Crick et al. reported that superhydrophobic surfaces fabricated from a silicone elastomer using aerosol-assisted chemical vapor deposition significantly reduce the counts of *Escherichia coli* (*E. coli*) and Methicillin-resistant *Staphylococcus aureus* (MRSA) [76]. In addition, Pernites et al. deposited polystyrene particles deposited on a surface and further modifying the surface with conductive polythiophene electropolymerization. The superhydrophobic surface demonstrated resistance to Gram-negative *E. coli* binding [77].

As aforementioned, surface-free energy and surface roughness should be taken into account in restraining bacterial adhesion. When in contact with the patterned surface, the bacteria cell membrane suspends between the interspacing of the micro/nanostructures. This stretching experienced eventually ruptures the membrane. Nano-textured surfaces with a high height-to-width-aspect ratio exhibit higher cell adhesion strength. The majority of previous studies indicated that superhydrophobic surface endows greater resistance towards attachment of Gram-negative bacteria than that of Gram-positive bacteria. This phenomenon may be due to the structure difference between Gram-negative and Gram-positive bacterial cells. The cell wall of Gram-positive bacteria consists of a thick, multi-layered peptidoglycan structure, thereby allowing Gram-positive bacteria to possess higher resistance to physical disruption, as compared to that of Gram-negative bacteria [78]. Interestingly, Gram-positive bacteria cells, including *Staphylococcus epidermidis* and *Staphylococcus aureus*, are more frequently found in indwelling medical devices-related infection as they are the most common commensal bacteria present on human skin and mucous membranes (refer to Table 3) [79]. However, the adhesion and growth of *Staphylococcus epidermidis* are greater on a rough surface, as compared to *Staphylococcus aureus*, who attach preferably on a smooth surface [80,81]. In view of this, different bacteria cells interact differently on a superhydrophobic surface. Therefore, further investigation is needed to ameliorate the antimicrobial function of superhydrophobic surfaces with respect to different bacteria cells.

## 5. Recent Superhydrophobic Modification on Medical Devices

Bark Jr. et al. modified a mechanical heart valve by spraying a superhydrophobic hierarchical coating on the medical device. The micro/nanoscale hierarchical texture is reported to exhibit a significantly low roll-off angle by increasing hydrophobicity, and is therefore able to repel blood. The modified surface of the mechanical heart valve creates a fluid slip along the surface, therefore altering the hemodynamic performance. It minimizes blood cell adhesion without inflicting damages to the blood. The report suggested that the superhydrophobic surface reduces shear stress by drag reduction, therefore lowering the energy dissipation across the device [3].

Similarly, Tan et al. further complemented the surface modification of cardiovascular devices by introducing a type of catecholamine, poly-norepinephrine [84]. The poly-norepinephrine coating is fabricated to act as a strategy to overcome the thrombotic complication in cardiovascular devices, such as stents and grafts. The multifunctional coating demonstrates low adhesion and activation of platelet cells, thus indicating an anti-thrombotic effect. To top it off, catecholamine coating helps to promote the proliferation of endothelial cells due to its selectivity of vascular cells, thereby accelerating the regeneration of the vascular cells. Despite the low apparent contact angle, the coating exhibits a low hemolysis rate (<1.5%). However, the hemocompatibility of this catecholamine coating is yet to be profoundly investigated.

Li and colleagues designed a hemostatic gauze by using immobilized carbon nanofiber (CNF) [35]. The CNF coating was fabricated by mixing CNF with polytetrafluoroethylene (PTFE) powder and polydimethylsiloxane (PDMS) respectively. The CNF/PTFE and CNF/PDMS composite were spray-coated on cotton woven gauze. Both the CNF/PTFE coated surface and CNF/PDMS coated surface exhibited a blood contact angle of 153.6° and 151.4°, respectively. The superhydrophobic surface is capable of withstanding substantial blood pressure, and therefore preventing blood loss. In addition, the superhydrophobic CNF-coated gauze demonstrated low bacterial adhesion, attributed to its low surface energy and rough texture. Interestingly, the superhydrophobic gauze is also reported to promote blood clotting by promoting fibrin fibers generation. The formed clots will eventually detach themselves easily. This self-cleaning effect is elucidated by the Cassie–Baxter state, indicating the presence of air pockets at the blood–substrate interface. These features and characteristics demonstrated the effectiveness of CNF as a hemostatic material.

Device-related infection remains a long-term challenge in the healthcare setting due to the stubbornness of biofilms. The development of biofilm facilitates the dispersion of the pathogenic bacteria into the other parts of the devices. The spreading of bacteria cells to the host’s body may lead to chronic infection and further complications. In light of this, medical devices, particularly invasive devices which are persistently in contact with the patient’s blood, require exquisite attention to hamper the formation of biofilm. Leslie et al. coated tethered-liquid perfluorocarbon (TLP) on tubing and catheters [57]. TLP coating roughened the surface with liquid perfluorocarbon. The resulting surface demonstrated superhydrophobic properties by exhibiting a sliding angle of less than 3°. The medical-grade perfluorocarbon-coated surfaces resisted adhesion of fibrin and platelets. Besides, the TLP coating suppressed clot formation and reduced thrombosis under blood flow. In addition to the blood repellence effect, the coating inhibits the formation of biofilm as well.

Corresponding to that of TLP-coated tubing and catheter, Ohko et al. fabricated a self-cleaning silicone catheter and medical tubes by coating titanium dioxide (TiO_2_) on them [85]. The TiO_2_-coated silicone catheters demonstrated significant antimicrobial properties, particularly against the Gram-negative *Escherichia coli* (*E. Coli)*. Besides, the TiO_2_ coating allows facile self-sterilization via UV illumination attributed to its photocatalytic properties. Albeit further investigation is required to improve the hydrophobicity of the coated catheter, their bactericidal effect and self-sterilizing properties provide insight into the fabrication of blood-compatible superhydrophobic coatings on medical devices. Silane treatment is substantiated to be a strategy that enhances the hydrophobicity, by not only lowering the surface-free energy, but also helps to improve the long-term stability of the coating [86,87].

In recent years, metals or non-metals nanoparticles have been extensively reviewed to be assimilated into superhydrophobic surface or biomaterial manufacturing. Metals that possess antibacterial properties in nature have been hybridized with other polymers to induce a synergistic effect on the blood compatibility of the medical devices. Recently, Tu et al. modified a medical tubing by applying a copper-phenolic-amine coating [88]. The remarkable anticoagulation effect attributed to the metal-chelating coating demonstrated a promising strategy to alleviate the medical device-associated thrombosis. Besides, the antibacterial effect of these blood-compatible coatings had been indicated via in vivo and in vitro studies. Besides, the polyurethane-gold-polyethylene glycol (PU-Au-PEG) hybrid composite mesh developed by Zhao et al. is reported to exhibit significant resistance towards the adhesion of bacterial cells. The blood-compatible surface of the mesh is suitable to be employed in hernia repairing, as the in vivo bactericidal performance of the surface on the infected hernia model has been validated. Moreover, the mesh does not induce damage to red blood cells while generating neglectable toxicity [89].

On the other hand, graphene is another promising biomaterial as a superhydrophobic surface or coating on blood-contacting medical devices. Graphene is a two-dimensional sheet of sp2 hybridized carbon in a honeycomb structure. Graphene surface tends to be positively charged and helps to improve surface hydrophobicity by reducing surface-free energy [90]. Geng et al. deposited graphene coating on a germanium surface via atmospheric pressure chemical vapor deposition (APCVD) [91]. The hemolysis rate of graphene upon contacting with fresh blood was significantly low. Besides, the bactericidal activities of graphene coatings have been reported. The mechanisms can be elucidated via physical damage and oxidative stress [92]. It should also be noted that the graphene coatings can destroy the membrane of bacteria via electrostatic interaction as well. However, platelet adhesion and activation were observed on the graphene surface due to the noncovalent interaction [91]. Another study implied the significance of graphene coating on cotton fabric. In addition to the primary surface roughness provided by the microscale cellulose fiber on the cotton fabric, the electric conductive graphene offers low surface-free energy. Upon supplemented with a secondary nanoscale roughness via silane treatment, the superhydrophobic properties are further enhanced [93]. Graphene was speculated to be utilized as a hemostatic material as well as the potential for constructing other medical applications such as thrombin protein detection biosensors.

## 6. Conclusions and Future Outlook

The development of biomaterials has been intense in recent years. Among other approaches, superhydrophobic properties are believed to be the current trend in developing a blood-repellent biomaterial. Superhydrophobic is described as a surface with high apparent contact angle, low surface energy, and high surface roughness. The roles of superhydrophobic surfaces in mitigating the long-term issue of hemocompatibility have been demonstrated, albeit enhancement is required to strengthen their effect. Superhydrophobic surface impedes hemolysis and alleviates thrombogenicity. In addition, the antifouling properties of superhydrophobic surfaces prevent device-related infection by reducing the adhesion of bacterial and other organic substances. These contributions of superhydrophobic surface thereof are postulated to act as a potential solution for the existing issue associated with the application of blood-contacting medical devices.

Studies in the future should focus on the blood–surface interactions in the presence of blood flow. The factors affecting the superhydrophobic characteristics, including surface morphology and surface chemical composition, require further investigation. The surface chemical compositions possess charges which exert different antiadhesive effects across various bacteria. Besides, the synergistic effect of superhydrophobic materials should be evaluated to produce an absolute blood-compatible biomaterial. Durability and sustainability of the superhydrophobic surface should also be a future prospect. The effectiveness of superhydrophobic surfaces over time and prolonged usage remains a question. Therefore, the physical and mechanical robustness of the superhydrophobic surface requires further study.

From the perspective of the antimicrobial effect of superhydrophobic surfaces, the underlying mechanism of biofilm formation is yet to be discovered. The initial adherence event of bacterial cells to the surface does not offer a significant impact on the thickness and amount of the biofilm formation. The likelihood of biofilm formation depends on the cell-to-cell interaction rather than the initial amount of cell-to-surface adherence. Therefore, further investigation is required to study the probability of the occurrence of device-associated infections and their virulency succeeding the introduction of superhydrophobic surfaces in blood-contacting medical devices. Besides, different bacterial cells react in a dissimilar way towards the superhydrophobic surface as their adherence to the surface is influenced by several factors. Therefore, further study is required to investigate this matter.

Importantly, an ideal multifunctional superhydrophobic surface or material is yet to be discovered. Various approaches to fabricate an effective and high-quality superhydrophobic surface have been reported. For instance, femtosecond laser ablation and electrospinning, with the help of modern technology, provides a desirable and uniform structured surface. Modification of the surface chemical composition by introducing hydrophobic and low surface energy nanoparticles and polymers showed promising results. Most of the approaches discussed herein are tedious and complicated. Among them, applications of superhydrophobic coating are frequently observed in the current approach due to their facile fabrication. However, their robustness, sustainability, and cytotoxicity are yet to be confirmed. Therefore, an economical and easy-to-replicate method to fabricate a practically ideal superhydrophobic surface requires further investigation.

## Figures and Tables

**Table 1:** Examples of superhydrophobic surfaces present in nature.

	Contact Angle (°)	References
Plant		
Lotus leaf (*Nelumbo nucifera*)	162	[24]
Rice leaf (*Oryza sativa*)	157	[26]
Chinese watermelon	159	[26]
Lyme grass (*Leymus arenarius*)	161	[27]
Perfoliate knotweed (*Polygonum perroliatum*)	162	[26]
Ramee leaf (*Boehmeria nivea*)	164	[26]
Taro plant leaf (*Colocasia esculenta*)	164	[27]
Purple setcreasea (*Setcreasea purpurea*)	167	[26]
Insect		
Horsefly (*Tabanus chrysurus*) wings	156	[26]
Butterfly (*Parantica sita*) wings	161	[28]
Walker’s cicada (*Meimuna opalifera) wings*	165	[26]
Water strider legs	167.6	[29]

**Table 2:** Examples of artificial superhydrophobic surfaces.

Materials	Fabrication Process	Contact Angle (°)	References
Carbon nanofiber coating	Mixing of carbon nanofiber with polytetrafluoroethylene to form composite dispersion	162.1	[35]
Fluorinated polymer foam (Fluoropor)	Photoinitiated radical polymerization of fluorinated perfluoropolyether methacrylate and alcohol derivatives	163.7	[36]
Graphene	Reduced graphene oxide surface-treated with silane	157	[37]
Polystyrene film	Vacuum casting of polystyrene film on porous template	151	[38]
	Electrospinning of polystyrene film and modified with perfluorodecyltrichlorosilane vapor deposition	168	[39]
Polytetrafluoroethylene	Plasma etching treatment using argon and oxygen gases	171.4	[40]
Silicon dioxide	Mixing of silicon dioxide nanoparticles with poly(methyl methacrylate) to form a dispersion	163.3	[41]
Titanium	Adonization process and modified with chemical vapor deposition of (heptadecafluoro-1,1,2,2-tetrahydrodecyl)trichlorosilane	164	[42]

**Table 3:** Examples of medical device-related infection and their common causative bacteria [67,82,83].

Types of Medical Device-Related Infection	Causative Microorganisms
Central venous catheter infection	Gram-positive bacteria *Staphylococcus epidermidis* *Staphylococcus aureus* Gram-negative bacteria *Pseudomonas aeruginosa* *Klebsiella pneumoniae* *Enterococcus faecalis*
Mechanical heart valve infection	Gram-positive bacteria *Staphylococcus epidermidis* *Staphylococcus aureus* *Streptococcus* spp.
Other catheter-related bloodstream infection	Gram-positive bacteria *Staphylococcus epidermidis* *Staphylococcus aureus* *Coagulase-negative staphylococcus* *Streptococcus* spp. Gram-negative bacteria *Pseudomonas* spp. *Enterococcus* spp.
